# Rheumatoid arthritis: identifying and characterising polymorphisms using rat models

**DOI:** 10.1242/dmm.026435

**Published:** 2016-10-01

**Authors:** Anthony C. Y. Yau, Rikard Holmdahl

**Affiliations:** 1 Medical Inflammation Research, Department of Medical Biochemistry and Biophysics, Karolinska Institutet, SE-171 77 Stockholm, Sweden; 2 Southern Medical University, Guangzhou 510515, China

**Keywords:** Rat models, Rheumatoid arthritis, Genetics, Susceptibility genes, Chronic inflammation, Congenic mapping

## Abstract

Rheumatoid arthritis is a chronic inflammatory joint disorder characterised by erosive inflammation of the articular cartilage and by destruction of the synovial joints. It is regulated by both genetic and environmental factors, and, currently, there is no preventative treatment or cure for this disease. Genome-wide association studies have identified ∼100 new loci associated with rheumatoid arthritis, in addition to the already known locus within the major histocompatibility complex II region. However, together, these loci account for only a modest fraction of the genetic variance associated with this disease and very little is known about the pathogenic roles of most of the risk loci identified. Here, we discuss how rat models of rheumatoid arthritis are being used to detect quantitative trait loci that regulate different arthritic traits by genetic linkage analysis and to positionally clone the underlying causative genes using congenic strains. By isolating specific loci on a fixed genetic background, congenic strains overcome the challenges of genetic heterogeneity and environmental interactions associated with human studies. Most importantly, congenic strains allow functional experimental studies be performed to investigate the pathological consequences of natural genetic polymorphisms, as illustrated by the discovery of several major disease genes that contribute to arthritis in rats. We discuss how these advances have provided new biological insights into arthritis in humans.

## Abstract (precis)

**Summary:** Using experimental models of rheumatoid arthritis, natural genetic polymorphisms associated with arthritis are identified and characterised in rats, bringing in new biological insights into arthritis in humans.

## Introduction

**Fig. 1.**
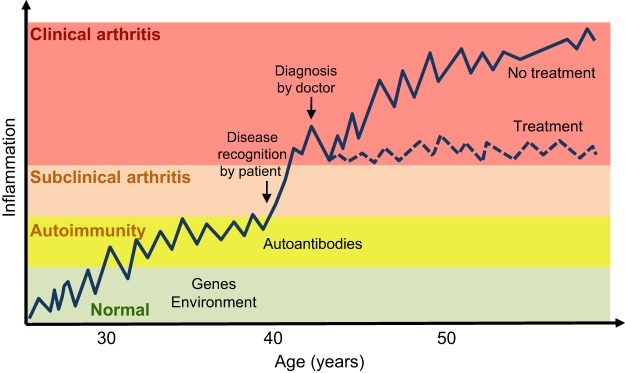
**The key stages of rheumatoid arthritis.** In this schematic, inflammation is plotted against the lifetime of an individual, who has or has not received treatment upon clinical diagnosis. The pathogenic autoimmune process passes through several stages. First, unknown environmental triggers (potentially including smoking, peridontisis and the gut microbiome) activate immune responses in genetically susceptible individuals many years before clinical onset. These responses can be identified by the production of autoantibodies, such as rheumatoid factor (RF) or anticitrullinated protein antibodies (ACPAs) in the serum. This stage is followed by a joint-specific inflammatory reaction, which leads to clinical onset and which can be perceived by individuals, leading to clinical diagnosis. In the last stage, the disease develops into a chronic phase.

Rheumatoid arthritis (RA) is characterised by chronic inflammation and by the destruction of synovial joints, leading to joint deformity and disability. It is more common in females, and affects around 0.5-1.0% of adults in the developed world (Scott et al., 2010). The pathogenic autoimmune process associated with RA consists of several distinct stages (Fig. 1) (Holmdahl et al., 2014). Initially, as-yet-unknown environmental triggers seem to activate innate immunity, inducing adaptive immune responses many years before clinical onset. These responses can be identified by the production of autoantibodies, such as rheumatoid factor (RF) or anticitrullinated protein antibodies (ACPAs), in serum (Aho et al., 1985, 2000; Brink et al., 2013; Rantapää-Dahlqvist et al., 2003). In time, a joint-specific inflammatory reaction occurs. This reaction is perceived by individuals and leads to clinical onset, and later to a clinical diagnosis. Finally, the disease develops into an active, chronic relapsing phase (Holmdahl et al., 2014).

Currently, there is no preventive treatment or cure for RA. The primary treatment is usually disease-modifying anti-rheumatic drugs (DMARDs), which reduce synovitis and systemic inflammation. Biological agents, such as antibodies that block tumour necrosis factor (TNF), have been used to treat RA patients who have failed to respond to treatment with conventional DMARDs (Keystone et al., 2004). Nevertheless, around one-third of anti-TNF-treated patients do not respond (Hetland et al., 2010; Klareskog et al., 2004). Therefore, one challenge in RA treatment is to identify the optimal therapy for each individual who is predisposed to developing RA or who has already developed the clinical disease. One approach is to develop potential biomarkers to predict the individual response to specific RA therapies, to allow future treatment to be based on an individual's genetic (Cui et al., 2013) and serological factors (Aho et al., 1985; Girbal-Neuhauser et al., 1997; Schellekens et al., 1998). Gaining a better understanding of the aetiology of RA is essential for developing therapies to cure this disease.

Although the cause of RA remains unclear, it is believed that both genetic and environmental factors contribute to its development and progression. One major environmental risk factor is smoking. Smoking increases the risk of developing classical RA, which is characterised by the presence of RF or ACPAs in the serum (Aletaha et al., 2010). Other potential environmental risk factors include a low alcohol intake and oral contraceptive use (Liao et al., 2009; Vessey et al., 1987).

The genetic contribution to RA is evident from the 15% concordance rate among monozygotic twin pairs (Silman et al., 1993). To date, genome-wide association studies (GWAS) of individuals with RA and healthy controls have identified ∼100 genetic loci that are linked to the disease (Okada et al., 2014). Human leukocyte antigen (HLA; see Glossary, Box 1) exhibits the strongest association to RA, with an odds ratio (OR; Box 1) of 2-3. The gene with the second strongest association to RA is the protein tyrosine phosphatase, non-receptor type 22 (*PTPN22*) gene (with an OR of 1.8). Most remaining risk loci are of modest effect (OR<1.3). HLA is estimated to account for ∼13% of the genetic risk to RA, whereas the other 100 loci account for another 5% (Okada et al., 2014; Raychaudhuri et al., 2012), which means that much of the total genetic contribution to RA remains unexplained. The identification of RA-predisposing genetic factors has been hampered by several factors, including genetic heterogeneity (Box 1), the low and variable penetrance of disease alleles (Box 1), linkage disequilibrium (Box 1) with nearby genes, and the possibility of gene–gene and gene–environment interactions (Okada et al., 2014).

**Box 1. Glossary**

**Adoptive transfer:** transfer of cells from a donor to a host.

**Advanced intercross lines (AILs):** lines generated by first crossing two inbred strains to produce an F1 generation and then subsequently intercrossing this generation and their progeny for many generations. These lines are used for linkage analysis to identify genetic regions that segregate with a disease or phenotype.

**F1 backcross:** after crossing two inbred strains to generate F1 hybrids, the F1 hybrids then mate with one of the two parental inbred strains. These lines are used in genetic linkage studies to identify genetic regions, usually large in size, that segregate with a disease or phenotype.

**F2 intercross:** the second-generation descendants of a cross of two inbred strains. The crossing of two inbred strains generates F1 hybrids, and these F1 hybrids mate with each other to produce F2 hybrids. These lines are used in genetic linkage studies to identify genetic regions, usually large in size, that segregate with a disease or phenotype.

**Genetic heterogeneity:** a phenomenon in which a single phenotype may be caused by multiple mutations or polymorphisms.

**Genetic** **linkage analysis:** an approach used to detect regions of the genome that contain gene(s) that predispose to a phenotypic trait by identifying genetic markers that co-segregate with the phenotype.

**Haplotype:** a group of closely linked genes that are inherited together on a single chromosome.

**Heterogeneous stock (HS):** stock generated by intercrossing several inbred strains for many generations. These lines are used for linkage analysis to identify smaller genetic regions that segregate with a disease or phenotype.

**Human leukocyte antigen (HLA):** the human gene complex that encodes the major histocompatibility complex. HLA-DRB1, encoded by the *HLA-DRB1* gene, forms the beta chain of membrane-bound HLA heterodimers, which present antigens to T helper cells.

**Linkage disequilibrium (LD):** the non-random association of alleles at different loci. The genotypes at the two loci are thus not independent of each other.

**Major histocompatibility complex (MHC):** heterodimeric membrane protein on the cell surface that helps the immune system to recognise foreign antigens by displaying peptides for T-cell recognition. Called human leukocyte antigen (HLA) in humans.

**Odds ratio (OR):** the odds that an outcome will occur given exposure to a particular factor (compared to the odds of an outcome occurring in the absence of that exposure). Such factors can be environmental or genetic (including genetic variants linked to disease). The OR can be calculated for cases compared to controls.

**Penetrance:** the proportion of individuals with a specific genotype that also expresses an associated trait (such as a disease).

**Positional cloning:** a method of gene identification in which a gene for a specific phenotype is identified only by its genomic location. Initially, linkage analysis identifies the approximate location of the genomic region concerned; positional cloning is then used to narrow this region until the gene associated with the specific phenotype is identified.

**Quantitative trait locus (QTL):** a genomic region linked to variation in a phenotype.

**Spontaneous mutations:** spontaneous genetic mutations can be induced in different ways (by chemical mutagenesis or by genetic means). Mutated animals are then screened for novel phenotypes. Once a phenotype is identified, its genetic basis can be identified using congenic strains.

Animal models of RA provide an attractive alternative approach to human genetics studies for identifying causative genes and to discover their underlying mechanisms. The use of these models in laboratory animals overcomes the challenges of genetic heterogeneity and environmental effects that feature in human studies. Animal models can also be used to identify disease loci, which can then be isolated on a fixed genetic background so that conclusive experiments can be performed to investigate specific disease pathways *in vivo* (Ahlqvist et al., 2011; Aitman et al., 2008; Baud et al., 2013; Vingsbo et al., 1996; Moreno-Moral and Petretto, 2016).

Over the past 20 or so years, several laboratories have been using different rat experimental models of RA to search for quantitative trait loci (QTLs; Box 1) that contribute to arthritis. Among the more than 100 arthritis QTLs identified in rats (see the Rat Genome Database, www.rgd.mcw.edu), five underlying causative genes or gene clusters have so far been successfully positionally cloned (Box 1). In this Review, using these five cloned genes as examples, we illustrate how rat models can be used to identify genes involved in the aetiology of arthritis and to advance our knowledge of the pathological functions of these genes. We also discuss how this approach complements other strategies available in both rodents and humans.

## Rat models of arthritis

Animal models of RA need to reflect the polygenic nature and environmental-factor-dependence of this disorder; they can also be used to model specific subsets of the disease. There are two categories of induced arthritis models in the rat: (1) disease induced by cartilage antigens, as exemplified by collagen-induced arthritis (CIA); and (2) disease induced by adjuvants alone, as exemplified by pristane-induced arthritis (PIA) (Vingsbo et al., 1996) or mineral-oil-induced arthritis (OIA) (Holmdahl et al., 1992a). The development of spontaneous arthritis in both rats and mice has been described as a result of genetic polymorphisms or mutations, for example in the genes *Ncf1* (neutrophil cytosolic factor 1) (Hultqvist et al., 2004) and *ZAP-70* [zeta-chain (TCR) associated protein kinase 70] (Sakaguchi et al., 2003), and as a result of environmental factors, such as the presence of commensal microbes (Wu et al., 2010) and hormonal and behavioural influences (Holmdahl et al., 1992b). Below, we describe the rat arthritis models in more detail.

### Collagen-induced arthritis

CIA is one of the most commonly used models of RA. In CIA, several different cartilage-derived proteins, including type II collagen (CII) (Trentham et al., 1977), type XI collagen (CXI) (Lu et al., 2002a) and cartilage oligomeric matrix protein (COMP) (Carlsén et al., 1998), are used to induce arthritis in rats, although almost all gene-mapping studies (discussed later) have been performed using CII-induced arthritis.

The CIA model is induced by injecting rats intradermally at the base of the tail with native autologous rat CII, emulsified in mineral oil [i.e. incomplete Freund's adjuvant (IFA)] (Holmdahl and Kvick, 1992). Importantly, CIA and OIA are different diseases but can be induced in the same rat strain (Holmdahl and Kvick, 1992). Rats of the Dark Agouti (DA) strain with CIA typically develop severe polyarthritis ∼2 weeks after immunisation, followed by a subsequent chronic relapsing phase (Holmdahl et al., 1992c, 1994). One important feature of CIA is the strong B-cell response specific for native CII epitopes (Holmdahl et al., 1994); these autoreactive B cells produce arthritogenic antibodies (Stuart et al., 1983). Another feature is the importance of autoreactive T cells, which are induced by immunisation with autologous CII (Goldschmidt and Holmdahl, 1991). However, the adoptive transfer (Box 1) of CIA by CII­-reactive T cells is not as effective as in the adjuvant-type arthritis models (Kleinau and Klareskog, 1993; Trentham et al., 1978), suggesting that the CIA model depends on both B and T cells in the rat. Similar to RA, both major histocompatibility complex (MHC; Box 1) and non-MHC genes contribute to CIA in the rat (Holmdahl et al., 1992c; Olofsson et al., 2003a; Remmers et al., 1996; Tuncel et al., 2012).

### Pristane-induced arthritis

PIA is another commonly used rat model of RA. PIA is a chronic relapsing arthritis model, induced by the injection of pure hydrocarbon oil pristane (2,6,10,14-tetramethylpentadecane) at the base of the tail (Tuncel et al., 2016; Vingsbo et al., 1996). Therefore, unlike CIA, PIA is independent of exogenously administered antigens. Despite being a natural component of plant chlorophyll, pristane injection leads to arthritis with disease onset around 10 days after injection in DA rats. The disease peaks around 20 days after injection; the inflammation then gradually disappears and is followed by relapsing chronic arthritis. PIA shares many clinical and subclinical features with RA (Haag et al., 2015; Lu et al., 2002b; Tuncel et al., 2016; Vingsbo et al., 1996). For instance, PIA develops symmetrically and affects peripheral joints. PIA rats are positive for RF and develop an acute-phase response. The mechanisms involved in the development of PIA remain unclear, but the disease is known to be αβ T-cell dependent (Holmdahl et al., 1992a), MHC class II (MHC-II) restricted, polyclonal and can be adoptively transferred (Box 1) by activated CD4^+^ T cells (Holmberg et al., 2006). Although innate immunity might be involved in driving inflammation in PIA (Hoffmann et al., 2011), there is as yet no evidence that B cells or antibodies have an arthritogenic role (Kleinau and Klareskog, 1993). PIA is regulated by both MHC and non-MHC loci (Olofsson et al., 2003b; Vingsbo et al., 1996).

### Oil-induced arthritis

Immunological adjuvants have been used for decades to enhance immunity to different antigens (Bomford, 1980; Freund, 1947). It was later discovered unexpectedly that injection of mineral oil as IFA induces arthritis in DA rats (Holmdahl et al., 1992a; Kleinau et al., 1991). Unlike PIA, which is induced by the defined alkane pristane, IFA is a poorly defined mixture of emulsifier and hydrocarbons of different molecular weights (Kuroda et al., 2004). In contrast to PIA, which induces a chronic, relapsing disease, OIA induces transient inflammation that subsides 2-3 weeks after onset (Holmdahl et al., 1992a). Other than these features, OIA is largely similar to PIA. It depends on the polyclonal activation of T cells (Kleinau and Klareskog, 1993) and on both MHC and non-MHC genes (Lorentzen et al., 1998).

### Other arthritis models

Other lipids, such as hexadecane, heptadecane and squalene, have been used to induce arthritis in rats, although they are not as commonly used as pristane and oil for genetic studies of RA (Carlson et al., 2000; Holm et al., 2001; Lorentzen, 1999). Another model used for gene analysis is mycobacterial adjuvant-induced arthritis (Mbt-AIA) (Joe et al., 2002; Kawahito et al., 1998). Mbt-AIA is monophasic and induced by the injection of complete Freund's adjuvant, which consists of both IFA and heat-killed mycobacteria. Although the pathogenic mechanism of Mbt-AIA is unclear, the heat shock protein 65 (HSP65)-derived peptide from mycobacteria is thought to be one of the immunogens of Mbt-AIA (van Eden et al., 1989). Components of the streptococcal cell wall have also been used to induce arthritis in rats in a model called streptococcal cell-wall-induced arthritis (SCWIA) (Cromartie et al., 1977).

## Different strategies of disease gene identification

**Fig. 2.**
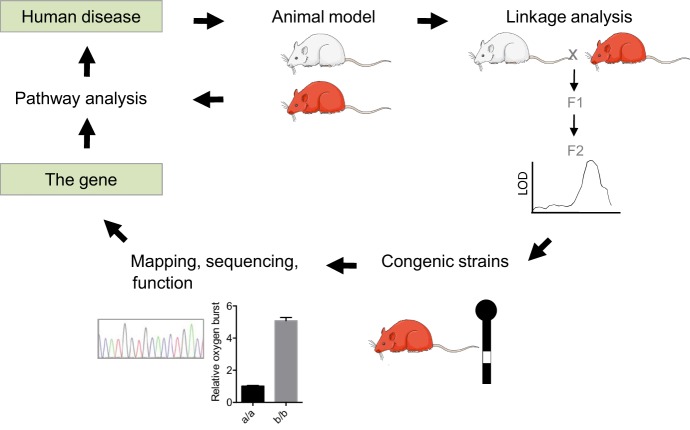
**Positional cloning of arthritis-linked genes in rats.** The identification of arthritis-associated genes using rat models involves several steps. First, genetic linkage analysis is performed. This identifies genetic markers that correlate with the arthritis trait that segregates in a population, leading to the identification of the quantitative trait locus (QTL) for that particular phenotype. The QTL concerned is then isolated in a congenic strain. The chromosomal region that associates with the phenotype is narrowed down to the smallest region possible, preferably containing only one gene, in a process known as positional cloning. Isolating a single gene in a congenic strain is usually not possible. Therefore, other methods, such as transgenesis, genetic engineering or functional assays, are used to identify the causative gene. Once the causative gene is identified, in-depth molecular pathway analysis and comparative studies with humans can be performed. Hypotheses generated from human studies can be tested in animal models to improve our understanding of the human disease. LOD, logarithm of odds.

**Fig. 3.**
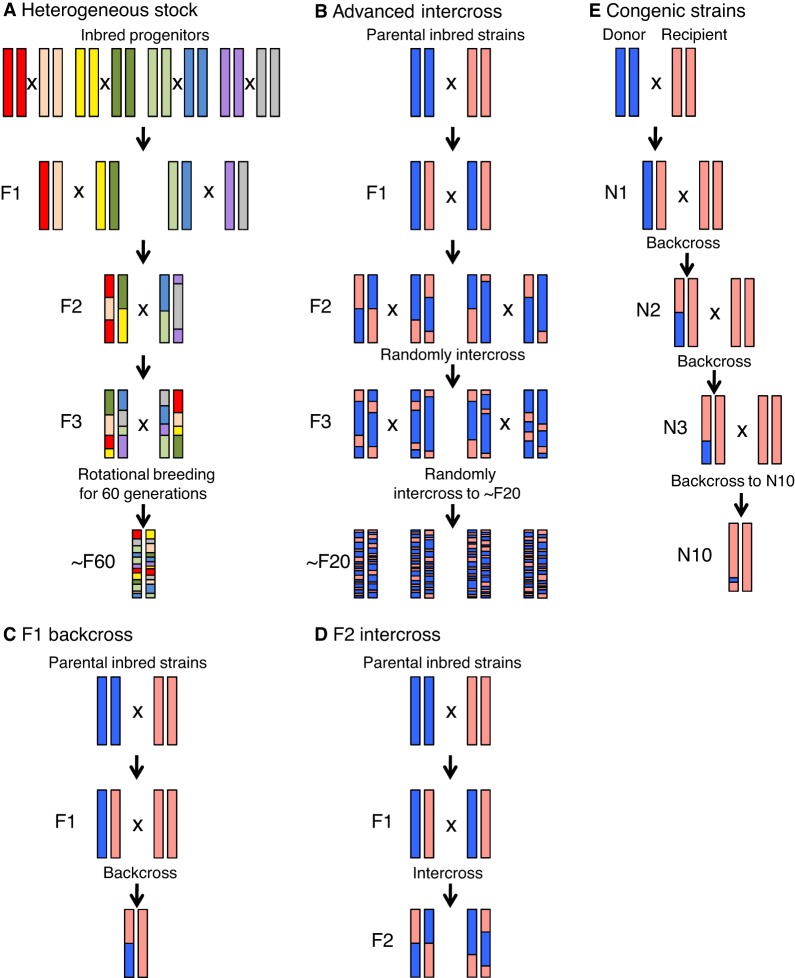
**Rat genetic lines used to study arthritis.** A schematic illustration of the generation of (A) heterogeneous stock, (B) advanced intercross lines, (C) F1 backcross, (D) F2 intercross and (E) congenic strains, which are used to identify candidate disease loci and genes in rats.

The availability of rat models of arthritis with stable and reproducible phenotypes facilitates genetic studies of this disease (Fig. 2). These studies employ different genome-wide mapping strategies, including F2 intercross, F1 backcross, advanced intercross lines (AILs), and heterogeneous stocks (HSs) (Box 1; Fig. 3). In mice, both AILs and HSs have been used for the high-resolution mapping of QTLs associated with different arthritis traits, including disease onset, severity, incidence and antibody production (Ahlqvist et al., 2011; Förster et al., 2012; Yu et al., 2006). HSs have also been developed in rats from eight inbred progenitor strains – ACI/N, BN/SsN, BUF/N, F344/N, M520/N, MR/N, WKY/N and WN/N (Johannesson et al., 2009) – and have been successfully used to map QTLs contributing to different phenotypic traits (Baud et al., 2013). However, these rats cannot be used to map arthritis-regulating QTLs because the inbred progenitor strains used to create them are resistant to the disease (J. Tuncel and R.H., unpublished).

Similar to the gene-mapping approaches in other diseases, the identification of arthritis-associated genes in rat models has typically involved several stages (Fig. 2). Initially, genetic linkage analysis (Box 1) is performed to identify the many different QTLs that regulate various arthritis phenotypes, including disease onset and severity, and also subphenotypes, such as CD4:CD8 T-cell ratio, and the presence of α­_1_-acid glycoprotein (a marker of the systemic inflammatory response) and of cartilage oligomeric matrix protein (a marker of cartilage destruction) (Kawahito et al., 1998; Lorentzen et al., 1998; Olofsson et al., 2003b; Remmers et al., 1996). The contribution of some of these QTLs to disease phenotypes has been reproduced in congenic strains (see Box 2) (Bäckdahl et al., 2003; Olofsson et al., 2003a; Remmers et al., 2002) and further analysed through the generation of smaller sub-congenic fragments, to narrow down the arthritis-associated loci (Haag et al., 2015; Lorentzen et al., 2007; Rintisch et al., 2010; Yau et al., 2016). The aim of this approach is to arrive at a genetic region that is linked to a disease but that contains a minimal number of genes, making it feasible to map and analyse different candidate genes and polymorphisms. These very small intervals may contain very closely linked genes as part of co-segregating gene clusters. The identification of causative genes or variants often requires an approach called positional cloning (Box 1), which relies on a phenotype being highly penetrant and on genetic recombination occurring between the causative locus and its nearest neighbours.

**Box 2. Generating and using congenic rat strains**

Congenic strains are generated by transferring a specific genetic locus from a donor strain to a recipient inbred strain. The first step involves crossing the donor strain to the recipient strain. The aim is then to replace, through multiple backcrosses, the ‘contaminating’ donor alleles with recipient alleles, except at the disease-associated locus. To achieve this, in each backcross, only offspring with donor alleles at the desired locus are selected for further breeding. Each backcross statistically reduces the ‘contaminating’ donor alleles by 50% and, after ten generations of backcrossing, the resulting new strain will statistically consist of ∼99.8% recipient strain. It is then considered to be a congenic strain. In many cases, congenic strains are generated to confirm the effect of QTLs identified in linkage analyses and, by dissecting the locus into smaller regions, to positionally clone the underlying causative gene. Therefore, once a congenic strain has been generated and the effect of the QTL has been reproduced in the congenic strain, attempts are made to minimise the size of the congenic fragment to preferably one or a few genes. At this point, the aim is to identify the causative gene(s) that regulates the phenotypic trait (such as a disease-relevant phenotype) and to study its functions.

Despite these challenges, five arthritis-associated genes or closely linked haplotypes (Box 1) have been positionally cloned from four QTLs to date, as illustrated in Fig. 3 and Table 1, and which we describe in more detail in the following sections. Most of these genes are not specific to arthritis and have been associated with other diseases as well. Among the five positionally cloned genes or haplotypes discussed in this Review, *MHC-II* genes were associated with RA in humans before these congenic studies, as discussed below. The generation of the *MHC-II* congenic strains supports a deeper analysis of the functions of these genes in arthritis. Other genes positionally identified from congenic studies, such as *Ncf1* and *APLEC*, were newly linked to arthritis, and have been investigated for their roles in RA in humans, as discussed below.

**Table 1.**
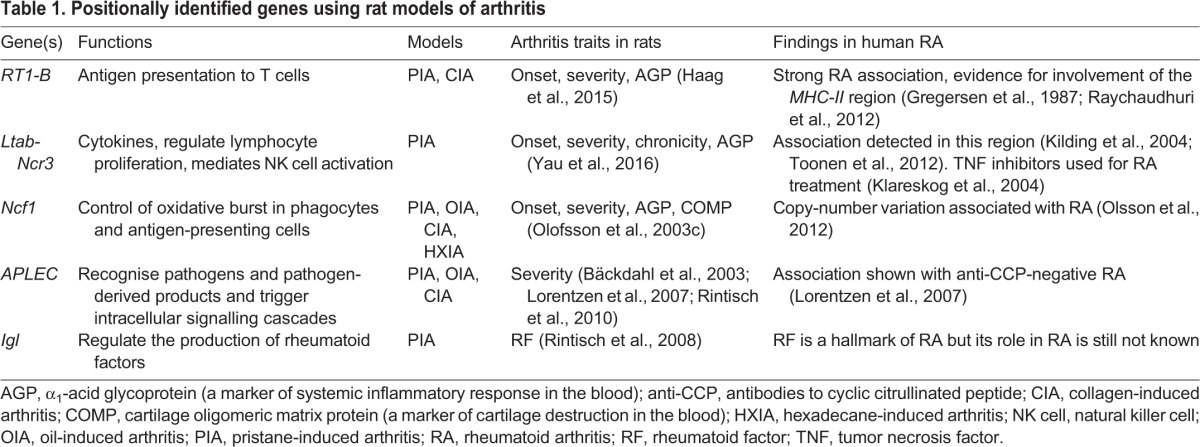
**Positionally identified genes using rat models of arthritis**

### Association with MHC-II genes

Although the association between RA and the MHC [called the human leukocyte antigen (HLA) in humans; Box 1) has been known for decades (Stastny, 1976), the underlying causative polymorphisms remain unknown. One theory – the shared epitope hypothesis – postulates that susceptibility to RA is associated with an epitope in the third hypervariable region of the HLA-DRb1 chain (Gregersen et al., 1987). This epitope was recently refined to amino acid positions 71 and 74, and extends to position 11 in individuals with ACPA-positive RA (Raychaudhuri et al., 2012).

In rats, the strong genetic association of MHC genes on chromosome 20 with susceptibility to different experimental arthritis models, including CIA, Mbt-AIA, PIA and OIA, was shown in different linkage analyses (Box 1) (Kawahito et al., 1998; Remmers et al., 1996; Vingsbo-Lundberg et al., 1998) and in MHC congenic strains (Griffiths and DeWitt, 1981, 1984; Lorentzen and Klareskog, 1996; Olofsson et al., 2003b; Remmers et al., 2002). The QTLs that contribute to arthritis in the CIA, Mbt-AI, PIA and OIA models are denoted *Cia1*, *Aia1*, *Pia1* and *Oia1*, respectively.

**Fig. 4.**
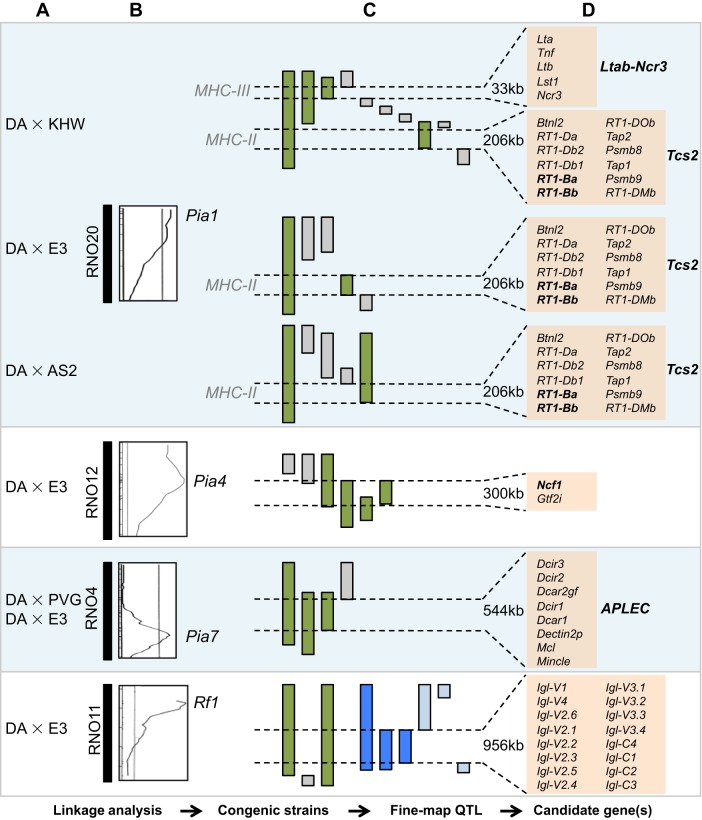
**The positional identification of genes underlying quantitative trait loci (QTLs) linked to arthritis using congenic rat strains.** (A) Inbred rat strains used for linkage analysis and/or construction of congenic strains. (B) Rat chromosomes are depicted as solid vertical blocks. On the right are exemplar logarithm of odds (LOD) likelihood plots for chromosome 20, 12 and 4 for clinical arthritis scoring, and for chromosome 11 for IgM rheumatoid factor (RF) (PIA, day 49). The lines inside the plots represent experiment-wide significance levels. The chromosomal regions with LOD above significance levels (*Pia1*, *Pia4*, *Pia7* and *Rf1*) were then further isolated and studied in congenic strains. (C) The genomic intervals of *Pia1*, *Pia4*, *Pia7* and *Rf1* QTL in the DA background were gradually narrowed through stepwise recombinations. For *Pia1*, *Pia4* and *Pia7*, the green bars denote PIA-protective/promoting strains, and grey bars denote strains with no effect on PIA. For *Rf1*, green bars denote congenic strains with RF phenotype; grey bars denote congenic strains with no RF phenotype; dark blue bars denote loci that correlate with RF-Igλ production in AIL strains (see Fig. 3); light blue bars denote loci with no RF-Igλ production. The location and size of genomic regions that link to arthritis and RF are indicated. (D) A list of genes in each arthritis- or RF-linked region. The genes in bold are linked to arthritis susceptibility. Note, not all of the congenic strains used in these studies are shown here, and maps of the congenic strains are not to scale. The data shown are derived from previous studies (Haag et al., 2015; Lorentzen et al., 2007; Olofsson and Holmdahl, 2003; Olofsson et al., 2003c; Rintisch et al., 2008; Tuncel et al., 2014; Wernhoff et al., 2003; Yau et al., 2016).

The association of the MHC-II region with arthritis has been further studied in a panel of *MHC-II* congenic strains that was generated by introgressing different inbred rat strains, namely KHW (called the RT1*^h^* haplotype), AS2 (RT1*^f^*) and E3 (RT1*^u^*) on a DA (RT1*^av1^*) background. These strains were used to map the arthritis severity effect previously shown for *Cia1* and *Pia1* to a 206-kb interval (called *Tcs2*) in the *MHC-II* region (Fig. 4) (Haag et al., 2015; Tuncel et al., 2012). In the PIA model, the *MHC-II* congenic strain with haplotype RT1*^f^* was associated with disease exacerbation, whereas the RT1*^h^* and RT1*^u^* haplotypes were associated with disease protection (Haag et al., 2015). In order to identify the disease-associated MHC-II genes, *Tcs2* coding variants were correlated with disease severity. The rat orthologues of the HLA genes, *HLA-DQA* and *HLA-DQB*, called *RT1-Ba* and *RT1-Bb*, respectively, were found to be the main candidate genes determining arthritis susceptibility. This was subsequently confirmed by the finding that treating both DA and congenic rats with an antibody against RT1-B resulted in a significant amelioration of arthritis (Haag et al., 2015). Further studies showed that the occupancy of the P1 pocket in the peptide-binding groove of RT1-B differs substantially between strains. In congenic rat strains that are protected from arthritis, this pocket binds preferentially to peptides with Glu, whereas, in disease-promoting congenic rat strains, it binds preferentially to hydrophobic residues, thus influencing the stability of RT1-B (Haag et al., 2015). In endosomes, the HLA protein, HLA-DM, catalyses the release of the class II-associated invariant chain peptides (CLIPs) from MHC-II in exchange for the loading of endosomal peptides onto MHC-II molecules; it is thus thought to be involved in ‘editing’ the peptide repertoire (Denzin and Cresswell, 1995; Kropshofer et al., 1996). One hypothesis is that the *RT1-B* coding variants could influence the susceptibility of *RT1-B* being edited by RT1-DM, thus contributing to the development of PIA (Haag et al., 2015).

The influence of *Tcs2* haplotypes on CIA has also been assessed, revealing that susceptibility to arthritis induced by the collagens CII and CXI is linked to haplotypes RT1*^av1^* and RT1*^f^*, respectively (Tuncel et al., 2012). Sequence analysis of the *MHC-II* genes has further shown that susceptibility to CIA is influenced by the *RT1-B* genes. In addition, in chronic PIA, rats with the RT1*^f^* haplotype develop strong cartilage-derived collagen reactivity that is specific to CXI and is restricted to RT1*^f^* MHC (Tuncel et al., 2012).

Studies of the MHC congenic strains have also shown that natural polymorphisms in another gene in the *MHC-II* region, called *Tap2*, contribute to the lineage commitment and negative selection of CD8 T cells, likely by altering the MHC-I peptide repertoire on antigen-presenting cells in the thymus (Tuncel et al., 2014). However, a role for this gene in arthritis or in any other disease remains to be investigated.

These studies in the rat were the first to directly compare different, naturally occurring *MHC-II* alleles on a fixed genetic background and to provide functional links between amino acid variants in an MHC-II molecule and the development of arthritis, as well as links to T-cell selection.

### Association with MHC-III genes

The MHC class III (*MHC-III*) region has also been independently linked to genetic susceptibility to RA (Kilding et al., 2004; Kimura et al., 2007; O'Rourke et al., 2008; Vignal et al., 2009). *MHC-III* is the most gene-dense region of the human genome and is located between *MHC-I* and *MHC-II* (Xie et al., 2003). The high gene density, extreme polymorphism and complex linkage disequilibrium across the MHC (Gabriel et al., 2002) complicate disease-association studies and make this region very difficult to investigate. As a result, evidence that variants in the *MHC-III* region really contribute to the pathogenesis of RA in humans and experimental models is lacking.

The association of the *MHC-III* region with arthritis has been studied using a panel of rat *MHC-III* congenic strains generated by introgressing different inbred rat strains, namely KHW (RT1*^h^* haplotype), AS2 (RT1*^f^*) and E3 (RT1*^u^*) on a DA (RT1*^av1^*) background, as with the previously discussed *MHC-II* congenic strains (Yau et al., 2016). This study showed that, in the *MHC* region, in addition to the *MHC-II* *RT1-B* genes (Haag et al., 2015), there is a second arthritis-associated QTL in *MHC-III* that influences the onset, severity and chronicity of arthritis (Fig. 4) (Yau et al., 2016). Unlike the *MHC-II* (*Tcs2*) locus, which is regulated by three haplotypes, this *MHC-III* locus is regulated by only one – the RT1*^h^* haplotype. This locus provides a protective effect, although not as strong as that of the *MHC-II* locus in acute PIA (Haag et al., 2015; Yau et al., 2016). By assessing PIA in different *MHC-III* congenic strains and by reducing the size of the arthritis QTL-containing region, the locus was fine-mapped to a 33-kb region in the telomeric end of *MHC-III*. This region contains five polymorphic genes, namely *Lta* (lymphotoxin-α), *Tnf* (tumour necrosis factor), *Ltb* (lymphotoxin-β), *Lst1* (leukocyte-specific transcript 1) and *Ncr3* (natural cytotoxicity-triggering receptor 3), which cluster together as a conserved haplotype (Yau et al., 2016). All five genes encode proteins with functions closely related to inflammation, and selective pressure might have conserved this haplotype, such that the variants in this cluster could operate in *cis*. In addition, higher *Ltb* and *Ncr3* expression, lower *Lst1* expression, and the expression of a shorter splice variant of *Lst1* correlate with reduced arthritis severity in PIA in the rat (Yau et al., 2016). This study illustrates that complex diseases such as arthritis can be regulated by haplotypes consisting of tightly linked genes.

### A protective role for Ncf1

In an F2 intercross between the arthritis-susceptible DA rat strain and the arthritis-resistant E3 strain, one of the strongest genetic associations to emerge in the PIA model was to the *Pia4* locus on chromosome 12 (Vingsbo-Lundberg et al., 1998). This association was subsequently confirmed in a genetic linkage analysis of an F1 cross (Olofsson et al., 2003b) and in the CIA model (Griffiths et al., 2000; Olofsson et al., 2003a).

In order to identify the susceptibility gene underlying this association, congenic strains with *Pia4* from the E3 genetic background were introgressed into the DA background and shown to display reduced arthritis severity (Olofsson et al., 2003c). To further dissect this region, a series of congenic strains were produced that contained smaller fragments of this region, and were examined for resistance to PIA. These congenic strains conclusively identified a minimal arthritis-protective interval of 300 kb that contained only two genes, *Ncf1* and *Gtf2i* (general transcription factor IIi) (Fig. 4) (Olofsson et al., 2003c). Neither gene is differentially expressed between the two rat strains, and only *Ncf1* contained non-synonymous single-nucleotide polymorphisms (SNPs) (at positions 106 and 153). *NCF1* encodes the p47^phox^ subunit of the phagocytic NADPH oxidase (NOX2) complex, which produces reactive oxygen species (ROS) (Volpp et al., 1989). The arthritis-protective congenic rat with an E3-derived *Ncf1* on a susceptible DA background showed an increased oxidative-burst response (Olofsson et al., 2003c). Further studies using a Wistar congenic strain that differs from DA at only position 153 in *Ncf1* conclusively showed that this position regulates ROS and mediates arthritis resistance in rats (Hultqvist et al., 2011). Mutational analysis of this SNP (substitution of methionine to threonine at position 153) demonstrated that this polymorphism did not affect the cytosolic assembly or the localisation of the NOX2 complex, but operates downstream of NOX2 assembly, thereby affecting the superoxide production of the NOX2 complex (Hultqvist et al., 2011). This discovery in rats was strengthened by the finding that a spontaneous mutation (Box 1) in the mouse *Ncf1* gene, which reduces *Ncf1* expression and produces an undetectable ROS response, gives rise to enhanced arthritis (Hultqvist et al., 2004). In humans, the *NCF1* region is characterised by deletions, duplications and inversions (Görlach et al., 1997). A case-control study has reported that an increase in *NCF1* copy number can protect against the development of RA (Olsson et al., 2012).

The above findings indicate that high levels of ROS reduce rather than increase inflammation. This is surprising because the release of ROS is widely believed to be proinflammatory. It was later shown that macrophage-derived ROS in particular play an important role in suppressing T-cell responses and arthritis severity (Gelderman et al., 2007). A lower capacity to produce ROS is associated with more thiol groups (-SH) on the T-cell membrane and most likely within the T-cell receptor (TCR) signalling complex, which increases T-cell activation and proliferation, and thereby determines T-cell arthritogenicity (Gelderman et al., 2006). *Ncf1* polymorphisms seem to be crucial for different inflammatory diseases, but the downstream mechanisms that induce oxidation are complex and operate in unique ways in different cells and in different diseases. For example, mutated *Ncf1* has been associated with the spontaneous development of lupus (Kelkka et al., 2014) and with increased severity of psoriasis in animal models (Khmaladze et al., 2014) and of gout in the mouse (Schauer et al., 2014). Our current understanding of the role of Ncf1 in autoimmunity has recently been reviewed (Holmdahl et al., 2016).

### Association with the *APLEC* gene complex

A locus on rat chromosome 4 that is associated with the PIA (Nordquist et al., 2000; Vingsbo-Lundberg et al., 1998), OIA (Lorentzen et al., 1998) and CIA (Griffiths et al., 2000) models was first identified in F2 crosses, and its genetic contribution was subsequently reproduced in congenic strains (Bäckdahl et al., 2003; Olofsson et al., 2003a,b; Ribbhammar et al., 2003). It was then positionally mapped to a 544-kb interval that corresponds to an evolutionarily conserved gene complex, called the antigen-presenting lectin-like receptor complex (*APLEC*). This complex consists of lectin-like receptor genes, including *Mincle* (macrophage-inducible C-type lectin), *Mcl* (macrophage C-type lectin), *Dcar1* [dendritic cell (DC) activating receptor 1], *Dcir1-4* (DC immunoreceptor 1-4) and the Dectin pseudogene *Dectin2p* (DC-associated C-type lectin 2 pseudogene) (Fig. 4) (Lorentzen et al., 2007; Rintisch et al., 2010). Adoptive transfer experiments performed in the *APLEC* congenic and DA rats showed that the *APLEC* locus controlled the priming of arthritogenic T cells and not the effector phase (Rintisch et al., 2010). Six of the seven genes in this complex are differentially expressed in the lymph node and/or carry a missense or nonsense mutation in DA rats (Lorentzen et al., 2007). It is currently unclear whether the effect of this locus is due to a haplotype, similar to that of the *Ltab-Ncr3* locus, with contributions coming from several interacting genetic variants, or is due to a single gene.

The *APLEC*-encoded receptors are type-II transmembrane proteins that are mainly expressed on neutrophils and antigen-presenting cells (APCs). The precise roles and functions of these genes are not completely understood. The structure of these receptors indicates that they have both inhibitory and activating signalling functions (Sancho and Reis e Sousa, 2012). For example, *Dcir1* and *Dcir2* signal through immunoreceptor tyrosine-based inhibitory motifs (ITIMs) in their cytoplasmic domains and play an inhibitory role (Kanazawa et al., 2002), whereas *Mincle* has an activating function through its immunoreceptor tyrosine-based activating motifs (ITAMs) (Yamasaki et al., 2008).

The *APLEC* gene cluster has also been implicated in other disease models, including MOG (myelin oligodendrocyte glycoprotein)-induced experimental autoimmune encephalomyelitis (EAE) (Flytzani et al., 2013), where it plays a putative role in disease susceptibility and severity, and antibody response. The *APLEC* gene cluster is also associated with motor neuron survival after traumatic nerve root injury (Lindblom et al., 2013). In knockout mice, these different receptor genes have been linked to the development of arthritis (Fujikado et al., 2008), to experimental colitis (Hütter et al., 2014; Tokieda et al., 2015) and to EAE, as well as being involved in the response to infection (Uto et al., 2016). Further studies are needed to better understand how the *APLEC* region is involved in immune regulation and in inflammation in humans.

### Rheumatoid factors

RFs were the first autoantibodies identified in RA and are still used to classify the disease (Aletaha et al., 2010). RFs are antibodies that recognise the Fc portion of immunoglobulin G (IgG). The presence of RFs in serum predates the onset of RA by several years (Rantapää-Dahlqvist et al., 2003), and positivity for RFs is associated with a better response to B-cell depletion therapy by rituximab (Chatzidionysiou et al., 2011) and to inhibition of T-cell co-stimulation therapy by abatacept (Gottenberg et al., 2016). However, relatively little is known about the genetic control of RFs.

In rats, linkage analysis on a (DA×E3) F_2_ cohort identified three genetic loci that regulate RF production (*Rf1*, *Rf2*, *Rf3*) (Wernhoff et al., 2003). To investigate the *Rf1* locus on chromosome 11, a 6.7-Mb congenic strain was generated by introgressing *Rf1* from E3 onto the DA background; this strain developed significantly elevated levels of RFs (Rintisch et al., 2008). Because this region undergoes little recombination, only two smaller subcongenic fragments (up to 3.2 Mb and 4.6 Mb, respectively) could be generated, which did not conclusively identify any genes that regulate RF production. To overcome this problem, the advanced intercross line (AIL) approach was used. After 19 to 21 generations of intercrossing GK and F344 rat strains (which have similar RF production patterns as that of the congenic and DA strain), the AIL rats were genotyped and analysed for RF levels. By combining data from both the congenic and AIL crosses, the study identified the *Igl* (immunoglobulin lambda light chain) locus as being responsible for the RF phenotype (Fig. 4) (Rintisch et al., 2008).

This congenic strain was also used to investigate the effect of RF-Igλ (RF of the immunoglobulin lambda light chain) on other inflammatory disease models. In a model for allergic bronchitis or asthma, the congenic rats developed more severe ovalbumin-induced airway inflammation (Rintisch et al., 2008). There was, however, no significant difference in the development of arthritis in the PIA model in congenic and DA rats. It is possible that the type of RF associated with the lambda gene is not directly involved in the development of T-cell-mediated PIA. Instead, it could be involved in other disease models that are antibody dependent, such as CIA. This study also clearly showed that RFs are not arthritis specific. In fact, RFs have been linked to disease severity in individuals with asthma (Kobayashi et al., 2004), and is also detected in other inflammatory diseases, such as primary Sjögren's syndrome (Markusse et al., 1993) and systemic lupus erythematosus (Witte et al., 2000), highlighting the pathological importance of RFs in the inflammatory process.

## Rat arthritis models: lessons learned and limitations

The purpose of performing different genetic and functional studies in rats is to advance our understanding of human disease and to translate this information into better therapies for patients. This Review highlights five examples of natural genetic polymorphisms that contribute to autoimmune arthritis, identified by positional cloning using rat congenic strains. These examples offer important insights into the pathogenesis of arthritis.

The main advantage of using experimental disease models, such as the rat, is that these models allow *in vivo* functional experiments to be performed in well-controlled genetic and environmental settings, which is not possible in humans. In particular, the rat provides a unique possibility to study arthritis induced by oil adjuvants, since adjuvants alone do not induce models of rheumatoid arthritis in other animal species, such as the mouse. It should be noted that high, iterated doses of pristane given intraperitoneally induce a severe inflammatory disease in mice that also involves joints, but it is different from PIA in rats and does not mimic RA (Hopkins et al., 1984). Mineral oil is one of the environmental risk factors of RA in humans; exposure has been associated with an increasing risk of developing the disease (Sverdrup et al., 2005). What exactly drives the priming of T cells in RA in humans is not known, and adjuvants, which are present in our environment in food, tobacco and pollution, could be involved. PIA, a model based on an arthritogenic component discovered in mineral oil, pristane, is an excellent model for studying the effect of adjuvants. PIA is highly reproducible, with an almost 100% incidence and induces a chronic relapsing disease course that closely mimics RA (Tuncel et al., 2016; Vingsbo et al., 1996). PIA is highly dependent on T-cell activation and is mediated through the transfer of MHC-II-restricted T cells (Holmberg et al., 2006) and is therefore suitable for studying T-cell-mediated mechanisms of arthritis. CIA, induced through immunising rats or mice with various cartilage proteins, is more complex. In addition to sharing T-cell dependence with PIA, CIA has the additional influence of B cells, owing to the *in vivo* affinity of CII-specific antibodies for cartilage (Kraetsch et al., 2001). Therefore, CIA can be used to study the antibody-dependent mechanisms of arthritis. Most known arthritis-linked genes and loci associate with both PIA and CIA (Table 1), including the *RT1-B* (Haag et al., 2015; Tuncel et al., 2012), *Ncf1* (Olofsson et al., 2003c) and *APLEC* (Bäckdahl et al., 2003; Lorentzen et al., 2007; Rintisch et al., 2010) genes discussed in this Review. A few loci are implicated in only certain arthritis models. For instance, *Cia4* regulates PIA and OIA but not CIA, whereas *Cia6* regulates OIA but not PIA and CIA (Remmers et al., 2002), indicating specificity in the pathways in which some of these QTLs operate. Adjuvant models, such as PIA and OIA, therefore complement the classical antigen-induced arthritis models, such as CIA, for studying different aspects of RA in humans.

The possibility to isolate congenic fragments on a fixed background allows us to study natural genetic variants of interest, while controlling for other genetic and environmental factors. For instance, by assessing arthritis in a panel of congenic fragments that cover different parts of the *MHC* region, it was shown that the MHC locus *Pia1* consists of two sub-loci in *MHC-II* and *MHC-III* (Yau et al., 2016). In humans, it has been difficult to study arthritis associated with the *MHC-III* region due to the strong linkage disequilibrium within the *MHC* region (Vignal et al., 2009). Studying this complex in the context of a congenic animal overcomes this difficulty because the effect of the neighbouring classical MHC alleles, as well as that of other genes associated with the disease, are shared with the controls and can thus be ruled out. The analysis of congenic strains has also revealed another disease-associated gene, *NCF1*, which is usually excluded and not studied in GWAS owing to the complexity of the *NCF1* region in humans (Olofsson et al., 2003c; Olsson and Holmdahl, 2012). A similar approach was utilised to identify and characterise other disease-associated genes, including *RT1-B* (Haag et al., 2015), *RF-Igl* (Rintisch et al., 2008) and the *APLEC* genes (Lorentzen et al., 2007; Rintisch et al., 2010).

Once a disease gene(s) is identified, we can use congenic strains to perform a deeper analysis of the pathological roles of the gene. For example, *Ncf1* congenic studies have revealed an unexpected, protective role of ROS in autoimmunity. Such findings contrast with the prevailing view of the role of ROS in inflammatory diseases, and have since been confirmed in both mice and humans (Holmdahl et al., 2016). With the discovery that many autoimmunity loci associate with multiple autoimmune diseases (Richard-Miceli and Criswell, 2012), including RA (Begovich et al., 2004; Bottini et al., 2004), congenic strains have become a highly useful tool for investigating the contributions of RA-associated genes to other autoimmune diseases. As previously discussed, congenic strains have been used to show that the *Ncf1* and *APLEC* polymorphisms contribute to not only arthritis but also EAE (Flytzani et al., 2013; Hultqvist et al., 2004), and that *Igl* contributes to ovalbumin-induced airway inflammation (Rintisch et al., 2008).

However, it is also important to consider the differences between experimentally induced arthritis and RA in humans when interpreting findings from rat arthritis models. First, experimental arthritis and RA are induced via different routes. Experimental arthritis is induced by intradermal injection at the base of the tail, whereas, in humans, the disease trigger is not known but could possibly include airborne pollutants, food, cosmetics and so on. Different routes of administration are known to lead to different disease outcomes in rats (Tuncel et al., 2016). Second, rat arthritis models are induced by relatively well-defined agents, such as an adjuvant and/or a particular antigen, such as collagen, reflecting certain aspects of RA, such as T-cell pathways or antibody-dependent mechanisms. This is useful for investigating specific types of RA mechanisms, but the disease course in a particular model might not mirror the complex course of RA in humans, which could include a combination of different triggers. Last, it is important to take into account any genetic differences between humans and rats. For instance, in the rat genome, there is only one *Ncf1* gene. In the human genome, several identical copies of *NCF1* exist due to segmental duplications (Bayés et al., 2003), and an increased copy number of *NCF1* is associated with lowered susceptibility to RA in humans (Olsson et al., 2012). On the other hand, differences in genomic structure between humans and rats can sometimes be useful in research; for instance, in detecting association with arthritis traits in the *MHC-III* region. This discovery was enabled because the rat *Ltab-Ncr3* *MHC-III* region is much further away from the RA-associated classical MHC-I genes than it is in the human genome, and is therefore less affected by linkage disequilibrium in the *MHC* region (Hurt et al., 2004).

There are also drawbacks and limitations to using the congenic approach to study arthritis. First, the process of isolating and narrowing down the putative loci in congenic strains requires a lot of animal breeding and screening, and it can often take several years to positionally clone the underlying arthritis gene(s). Second, the success of positional cloning using congenic strains depends on identifying genetic recombinations. Both recombination-rich hotspots (regions with increased recombination over neighbouring regions) exist in genomes, as well as coldspots (Jensen-Seaman et al., 2004). Thus, some linked regions are very difficult to split by genetic recombination, and other approaches, such as the use of AILs, are needed to facilitate positional cloning (Rintisch et al., 2008). Nevertheless, obtaining conclusive evidence of the functional consequence of any particular polymorphism often requires additional genetic manipulation of the candidate genes.

Transgenic and knockout technology are typically used to characterise the functions of candidate genes. Previously, such technologies were limited to mice, but new advances have enabled scientists to modify the genes of any species, including rats. It is noteworthy that all genetic manipulations are vulnerable to artifacts and could lead to different phenotypic outcomes. For example, *Ncf1* knockout mice were reported to be completely protected from EAE (van der Veen et al., 2000), whereas naturally occurring *Ncf1* loss-of-function mutant mice developed enhanced EAE (Hultqvist et al., 2004). Female *Ncf1* knockout mice were protected from CIA, whereas female *Ncf1* mutant mice developed severe CIA (Sareila et al., 2015). These phenotypic differences can be due to the targeted gene being closely flanked by regions of donor origin in genetically modified mice despite repeated backcrossing to the host strain (Vanden Berghe et al., 2015). By contrast, in the congenic approach, instead of studying genetic variants selected and introduced by researchers, naturally occurring genetic variants are studied, which might be of greater relevance for understanding the pathways involved in human disease. Furthermore, although the targeted mutagenesis approach can be very useful for testing specific hypotheses, it does not explain variation in complex traits. Instead of being caused by a single sequence variant, phenotypic traits can be caused by QTLs that contribute effects from multiple causal variants within a single QTL (Baud et al., 2013, Moreno-Moral and Petretto, 2016), as could be the case in the *MHC-III*-region *Ltab-Nc3* gene cluster, which is a conserved haplotype found in both inbred and wild rats (Yau et al., 2016). Although the positional cloning and functional assessment of genes in animal models is both time-consuming and expensive, it is by far more cost-effective than performing this research in humans, for example, in large-scale GWAS.

## Future perspectives for RA

Over the past few decades, the scientific community has made tremendous progress in dissecting the complex genetics associated with RA, including the identification of over 100 risk loci by GWAS (Okada et al., 2014). It seems likely, both from the presented data and from evolutionary logic, that the pathways leading to arthritis in experimental animals and humans are shared, if not the exact same polymorphisms. It is therefore useful to study pathways involved in arthritis, and their associated genetic polymorphisms, in a relevant pathophysiological context. Thus, animal models are not only a helpful tool but also essential for understanding the pathways that lead to disease in humans. Both the genetic and environmental factors that contribute to arthritis can be investigated in animals. In addition, when it comes to the investigation of the exact causative polymorphism, it is possible to introduce a candidate causative human genetic polymorphism into an animal model, although care needs to be taken so that non-physiological genetic interactions are avoided, which requires a deep knowledge of animal complex genetics. Three challenges need to be overcome to make this a more efficient translational approach that can help us improve our knowledge of RA in humans.

First, the specific polymorphisms that underpin the GWAS-identified QTLs have not been conclusively identified; currently, the known risk loci account for less than one-fifth of the genetic risk of RA (Okada et al., 2014; Raychaudhuri et al., 2012). As we have discussed, rat models of RA can overcome some of the limitations of human studies. The congenic approach has been particularly fruitful for identifying previously unknown disease-associated genes, with more likely to follow. Part of the unexplained genetic variance in RA could in fact be due to epistatic interactions between different loci (both MHC and non-MHC). Constructing ‘double congenic’ lines to study interactions between MHC and non-MHC genes, and between non-MHC loci, could facilitate this endeavor. In addition, genes are subject to epigenetic regulation, and different epigenetic mechanisms, such as post-translational histone modifications and DNA methylation, play crucial roles in gene regulation, which may provide additional subtle contributions to RA susceptibility.

Second, we know very little about the pathogenic roles of most of the RA risk loci identified. We have discussed how congenic rat strains can be used to study the functional roles of different risk loci in rat arthritis models. Recently, the CRISPR/Cas system has emerged as a powerful strategy to generate rat strains with mutations in one or multiple genes (Li et al., 2013), which will be very useful for verifying and characterising different candidate genes *in vivo*. It is, however, important to bear in mind that such methods can, like other genetic manipulation technologies, introduce artifacts such as off-target mutations (Fu et al., 2013). In addition, such mutations will operate in the context of an inbred strain, and it is therefore important to understand the impact of this genetic context on the defined arthritis models being used. Thus, well-characterised disease models will remain essential for the identification and analysis of RA disease genes and pathways and for the validation of different therapeutic approaches.

Lastly, the environmental influence of RA is still unclear, and animal models are optimal for studying this. Environmental factors suspected to be of importance in human RA should be studied in a controlled way in animal models; for example, smoking, infections and the influence of mucosal bacteria, which can all present the immune system with a unique adjuvant exposure.
